# Case Report: Blinatumomab as upfront consolidation and maintenance therapy in a pediatric patient with high-risk B-cell acute lymphoblastic leukemia

**DOI:** 10.3389/fonc.2023.1246924

**Published:** 2023-11-01

**Authors:** Sumit Gupta, Jessica Casey, Joseph Lasky

**Affiliations:** ^1^ Department of Pediatric Hematology/Oncology, Cure 4 The Kids, Roseman University of Health Sciences, Las Vegas, NV, United States; ^2^ Department of Pediatrics, University of Nevada, Las Vegas, NV, United States

**Keywords:** blinatumomab, pediatric, B-cell acute lymphoblastic leukemia, maintenance therapy, case report

## Abstract

**Introduction:**

B-cell acute lymphoblastic leukemia (B-ALL) is the most common malignancy in children. The current conventional chemotherapy regimens have high overall survival but with significant short- and long-term toxicities, sometimes requiring delay and termination of chemotherapy. Bispecific T-cell engager antibody blinatumomab has been successful in achieving bone marrow remission and acting as bridging therapy in minimal residual disease (MRD)-positive relapsed adult and pediatric B-ALL patients. Its role as upfront therapy is being explored. Here, we report the first case to our knowledge showing the feasibility, tolerability, and sustained remission using blinatumomab upfront as consolidation and maintenance therapy for 2 years in a pediatric patient with high-risk B-ALL who had significant toxicities with conventional chemotherapy.

**'Case presentation:**

An 11-year-old Hispanic girl presented with complaints of fever, abdominal pain, and fatigue. On further evaluation, she had tachycardia, pallor, cervical lymphadenopathy, and pancytopenia. Bone marrow studies confirmed high-risk B-ALL. The patient was started on induction chemotherapy per AALL1131. Her induction course was complicated by syncope, febrile neutropenia, and invasive cryptococcal fungal infection. End-of-induction bone marrow results were MRD negative. Further chemotherapy was withheld due to cardiopulmonary and renal failure, along with ventricular arrhythmias requiring intensive care. The patient received two cycles of blinatumomab as consolidation therapy and then transitioned back to conventional consolidation therapy; however, it was terminated mid-consolidation due to Pseudomonas and Aspergillus sepsis. She was then given blinatumomab maintenance therapy for 2 years and tolerated it well without any irreversible toxicity. She had an episode of *Staphylococcus epidermidis* sepsis and pneumonia treated by antibiotics and a single episode of a seizure while on blinatumomab therapy. At the time of publication, she is 25 months off treatment and in sustained remission without any further transplant or chemotherapy. She received monthly intravenous immunoglobulin G during the blinatumomab maintenance.

**Conclusion:**

Blinatumomab given upfront as consolidation and maintenance therapy for 2 years in a pediatric high-risk B-ALL patient with significant toxicities to conventional chemotherapy was feasible and very well tolerated without any irreversible toxicity and led to sustained remission without any bridging transplant or further chemotherapy.

## 1 Introduction

Acute lymphoblastic leukemia is the most common malignancy in children with B-cell acute lymphoblastic leukemia (B-ALL), comprising 85% of leukemia cases (1). Over the decades, research through various clinical trials has resulted in increased survival using conventional chemotherapy, reaching up to 90% in some developed countries (2, 3). However, a significant number of toxicities are associated with these regimens leading to delay or interruption in chemotherapy, as well as short- and long-term morbidity and mortality (4). These range from mucositis, bacterial and invasive fungal infections, colitis, seizures, and osteonecrosis during the therapy to secondary malignancy, cardiotoxicity, obesity, endocrine abnormalities including hypogonadism, neurocognitive deficits, and premature death long-term (4–8).

Blinatumomab is a bispecific T-cell engager antibody construct that helps remove CD19-positive lymphoblasts by binding simultaneously to CD3-positive cytotoxic T cells (9). Based on clinical trials, it has been provided accelerated approval by the U.S. Food and drug administration (FDA) for use in Philadelphia chromosome-negative and minimal residual disease (MRD)-positive relapsed B-ALL patients, both in adults and in children (9, 10). Two major pediatric phase III trials for relapsed B-ALL, AALL1331 by Children’s oncology group (COG) and NCT02393859 in Europe, terminated enrollment prematurely due to significant beneficial results when used in consolidation for post-induction MRD positive relapsed B-ALL patients (10–12). The pediatric patients had fewer serious adverse events (SAE) as compared with conventional chemotherapy in these trials, and most of them tolerated blinatumomab well (11, 12). Current COG clinical trial AALL1731 is evaluating the effect of adding two cycles of blinatumomab upfront in standard risk-average B-ALL patients after consolidation. However, most adult and pediatric trials use blinatumomab for one to eight cycles as a bridging therapy to achieve remission and continue with either hematopoietic stem cell transplant (HSCT) or conventional chemotherapy (9, 11, 12). Here, we present the first case in the literature demonstrating the feasibility, tolerability, and treatment using blinatumomab upfront as consolidation (two cycles) and maintenance therapy (21 cycles) for 2 years in a pediatric patient with high-risk B-ALL who had significant toxicities, including multiorgan failure with conventional chemotherapy, leading to sustained remission without any further bridging therapy.

## 2 Case description

An 11-year-old Hispanic girl with no significant past medical history presented with high-grade fever, abdominal pain for 3 days, and decreased appetite and fatigue. She had no significant past medical and family history. On examination, she had tachycardia, pallor, and cervical lymphadenopathy. Laboratory findings were consistent with pancytopenia: WBC 1.3 × 10^9^/L, hemoglobin 7.4 g/dL, platelets 51 × 10^9^/L, and 3% neutrophils on differential. Chest X-ray revealed right perihilar and upper lobe infiltrates. Bone marrow (BM) and cerebrospinal fluid (CSF) studies confirmed B-ALL with 94% blasts (positive for CD19, CD10, CD20, CD22, CD34, CD38, CD79a, HLA-DR, and TDT) with CNS1 status. Cytogenetics studies revealed hyperdiploidy of multiple chromosomes (chromosomes 4, 5, 6, 10, 14, 17, 18, 21, and X) and fluorescence *in situ* hybridization studies confirming trisomy of chromosomes 4, 10, and 17 with two extra copies of RUNX1. The patient was started on a four-drug induction chemotherapy as per COG AALL1131. Induction chemotherapy was complicated by grade 2 hypertension, an episode of syncope, persistent febrile neutropenia, and fungemia with *Cryptococcus albidus* requiring antibiotics and antifungals (treated with amphotericin for 2 weeks and voriconazole that was continued until 6 months off blinatumomab therapy). CT chest/abdomen/pelvis revealed numerous lesions in the lungs, liver, spleen, and kidneys with biopsy of the hepatic lesion confirming disseminated fungal infection. End-of-induction BM confirmed an MRD-negative status. However, the patient had prolonged neutropenia and the course was further complicated by cardiorespiratory failure, renal failure, and symptomatic ventricular arrhythmias with Torsades de Pointes leading to intubation and intensive care, requiring 3 weeks to recover post-induction. She had generalized weakness, more profound in her bilateral lower extremities, requiring a wheelchair for mobility. Repeat BM aspiration 3 weeks post-induction showed an MRD-negative status.

**Figure 1:**
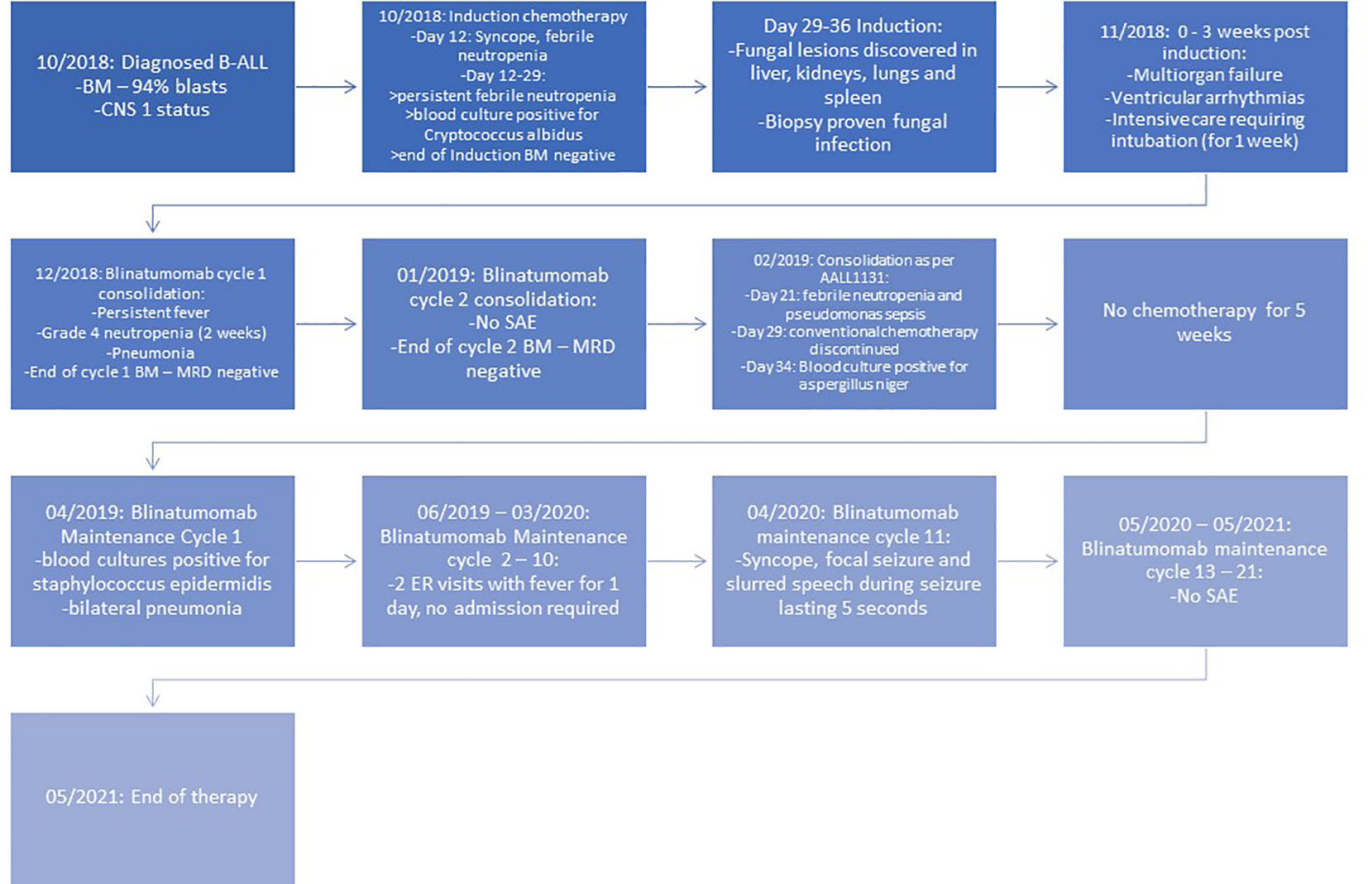
Timeline of events. BM, bone marrow; CNS, central nervous system; SAE, serious adverse event.

The patient was then started on blinatumomab consolidation (15 mcg/m^2^/day) for two cycles (28 days of continuous infusion followed by a 1-week break) after amphotericin was discontinued and received dexamethasone for 2 days during her first cycle due to persistent fevers. She had one episode of pneumonia treated with antibiotics during cycle 1. The patient received a lumbar puncture with intrathecal methotrexate on days 8 and 29 of each cycle. BM aspirate after each cycle of blinatumomab was performed and confirmed an MRD-negative status. Her generalized strength improved to the extent that she was able to sit independently. She was then transitioned back to conventional chemotherapy as per high-risk consolidation of COG AALL1131. Three weeks into the first half of consolidation chemotherapy, she presented with *Pseudomonas aeruginosa* sepsis. Further treatment was complicated by *Aspergillus niger* fungemia and prolonged myelosuppression, followed by the removal of her port and discontinuation of conventional chemotherapy mid-consolidation. Once recovered, she was started on blinatumomab maintenance immunotherapy (15 mcg/m^2^/day, each cycle of 35 days with 28 days of continuous infusion) that was continued for an additional 21 cycles to complete 2 years of treatment post-consolidation. She received two lumbar punctures with intrathecal chemotherapy each cycle for the first four cycles of maintenance therapy, followed by one dose of intrathecal chemotherapy per cycle for the remaining cycles. She developed *Staphylococcus epidermidis* sepsis requiring change of her PICC line and bilateral pneumonia during cycle 1. She also experienced an episode of syncope followed by self-resolving slurred speech and focal seizure (involving face and head) for 5 seconds during cycle 11 of blinatumomab maintenance. She was started on Keppra and continued to take Keppra. The patient was given monthly intravenous immunoglobulin G (IVIG) during and after completion of the treatment, which was eventually weaned to every 6 weeks. She tolerated blinatumomab therapy well and remains in sustained remission at 25 months off treatment at the time of publication, without any further transplant and chemotherapy. Her strength has improved dramatically. The timeline is shown in **Figure 1**.

## 3 Discussion

B-ALL is the most common type of leukemia diagnosed in children. The overall survival (OS) and event-free survival (EFS) have increased drastically following multiple successful clinical trials over the period of decades. However, subpopulations like National Cancer Institute (NCI) high-risk patients with unfavorable genetics or slow responders continue to have poor survival rates. The COG clinical trial AALL1131 tried to augment therapy with cyclophosphamide, etoposide, and clofarabine but showed high toxicity with no improvement in survival (1, 2, 13). Significant toxicities are associated with conventional chemotherapy (1, 5). In the COG clinical trial AALL0232, during induction around 20% and 11% of the patients had infections and febrile neutropenia respectively (5). High-dose methotrexate caused infections and mucositis in 12.3% and 14.4% patients, respectively, along with seizures and ischemic cerebrovascular events in 18 and 5 patients, respectively (5). Prolonged neutropenia, high-dose steroids, and chemotherapy also cause invasive fungal infections in as high as 8% of the patients (6, 14). These complications sometimes lead to the interruption of chemotherapy and contribute to both short- and long-term morbidity and premature mortality. The cumulative incidence for severe chronic medical conditions, including death, was 21.3% for survivors at 25 years from diagnosis (15). Current trials are investigating ways to reduce these toxicities while maintaining an excellent prognosis. Clinical trial AALL0932 aimed to reduce the toxicity by decreasing the pulses of dexamethasone and vincristine for the average risk subset of NCI standard risk B-ALL patients (5, 16). Our patient had significant toxicities, including grade 2 hypertension, invasive disseminated fungal infection during induction followed by cardiopulmonary and renal failure, along with ventricular arrhythmias, prolonged neutropenia, severe generalized weakness restricting mobility, and delay of chemotherapy post-induction.

Blinatumomab is a bispecific T-cell engager antibody construct that eliminates selective B lymphoblasts with CD19 markers using cytotoxic CD3+ T lymphocytes (9, 10). In adults, the phase III TOWER trial involved patients with Philadelphia negative MRD-positive relapsed B-ALL. Treatment with blinatumomab resulted in a higher rate of EFS compared with chemotherapy (31% vs. 12%) and longer duration of survival (9). It has also been shown to be superior to conventional chemotherapy in relapsed B-ALL pediatric patients. In the pediatric phase III COG AALL1331 study, relapsed B-ALL patients treated with two cycles of blinatumomab had a 2-year overall survival of 71.3% compared with 58.4% in the chemotherapy group, which led to the early termination of the trial (11). Another pediatric phase III multicentric trial in Europe used one cycle of blinatumomab for a third consolidation in relapsed B-ALL patients and showed that MRD remission was achieved in 90% vs. 54% of patients in the consolidation chemotherapy group. The incidence of events (relapse, second malignancy, death, or failure to achieve complete remission) was 31% vs. 57% in the blinatumomab group vs. the consolidation chemotherapy group (12).

Blinatumomab has been very well tolerated in children. In the COG trial AALL1331, the rate of SAE was lower in the blinatumomab group with cumulative rates of 15%, 5%, and 2% vs. 65%, 58%, and 27% in the chemotherapy group for infection, febrile neutropenia, and sepsis, respectively. The most common adverse event (AE) in the blinatumomab group was cytokine release syndrome (CRS) at 22% in cycle 1, but only 1% had equal or greater to grade 3 CRS. Encephalopathy and seizures were present in 11% and 4%, respectively, with 2% and 1% equal to or greater than grade 3. There were no AE-related deaths in the blinatumomab group, and all AEs were fully reversible (11). A similar safety profile was also seen in other pediatric trials (10, 12). Given the complications in our patient and the well-tolerated profile of blinatumomab, we decided to start consolidation with blinatumomab until she recovered from complications and invasive fungal infection. She had fevers consistent with grade 1 CRS in cycle 1 of blinatumomab, requiring dexamethasone for 2 days. However, she tolerated further cycles without any clinical evidence of CRS. Her fever could also be due to an underlying infectious process. She gained significant strength and was able to sit up independently by the end of cycle 2. We also examined the MRD status after each cycle of blinatumomab in consolidation, which continued to be negative.

All of the above clinical trials used blinatumomab for MRD-positive disease in relapsed B-ALL patients. However, the effect of blinatumomab when used upfront and in MRD-negative patients is also being studied both in adults and in pediatrics. In an adult phase III randomized national cooperative clinical trials network trial from ECOG-ACRIN E1910 involving 488 participants with newly diagnosed B-ALL, 224 patients that were MRD negative after the intensification of chemotherapy were randomized to receive four additional cycles of blinatumomab as part of their consolidation chemotherapy. Results showed that the upper boundary for efficacy analysis was crossed in favor of the blinatumomab arm with no significant safety concerns. It concluded that the addition of blinatumomab in consolidation for newly diagnosed B-ALL adults who are MRD negative at the end of intensification resulted in significantly better overall survival representing a new standard of care in ALL patients (17). The COG trial AALL1731 (NCT03914625) is determining if there is any improvement in overall survival when blinatumomab is given upfront for two cycles post-consolidation in the NCI standard-risk–avg B-ALL (MRD negative post-induction, but next-generation sequencing positive or indeterminate) and standard-risk–high B-ALL (end of induction or consolidation MRD <0.1%). Hence, we used blinatumomab for consolidation and maintenance therapy in our patient who had MRD negative bone marrow status at the end of induction.

In an adult multicentric study conducted in Europe involving relapsed B-ALL patients with MRD positivity after a minimum of three blocks of intensive therapy, 36 (33%) of the patients were not able to undergo hematopoietic stem cell transplant (HSCT). Out of these 36 patients, 9 (25%) remained in continuous remission without HSCT or chemotherapy after blinatumomab, with a median follow up of 24 months (18). Also, some patients required more than one cycle of blinatumomab to attain MRD remission (around 10% after the second cycle) (18). Similar results were also seen in other studies (19, 20).

Our patient had multiple episodes of sepsis, fungemia with disseminated invasive fungal infection, pneumonia, and prolonged myelosuppression leading to cardiopulmonary failure during the administration of conventional induction therapy and the first half of conventional consolidation chemotherapy. We used blinatumomab as consolidation therapy after induction to maintain remission given the high probability of relapse in high-risk B-ALL patients with delayed and interrupted chemotherapy. Once she recovered, we resumed conventional consolidation chemotherapy, which was complicated by *Pseudomonas aeruginosa* sepsis and *Aspergillus niger* fungemia. Hence, the decision was made to discontinue conventional chemotherapy mid-consolidation and use blinatumomab as further maintenance chemotherapy based on the safety profile, high risk of relapse without maintenance, and rationale as described in the above sections. There are no data in the literature regarding an appropriate duration of therapy with blinatumomab when it is not used as bridging therapy. The current or previous clinical trials used blinatumomab up to eight cycles in adults and two cycles in pediatrics when using it as a bridge (9, 11, 12). We gave blinatumomab maintenance for 21 cycles to complete 2 years of therapy after consolidation in line with the timeframe in most of the COG protocols for conventional chemotherapy in B-ALL. Based on COG protocol AALL1331, we gave 1 week off between two subsequent cycles of blinatumomab.

**Figure 2:**
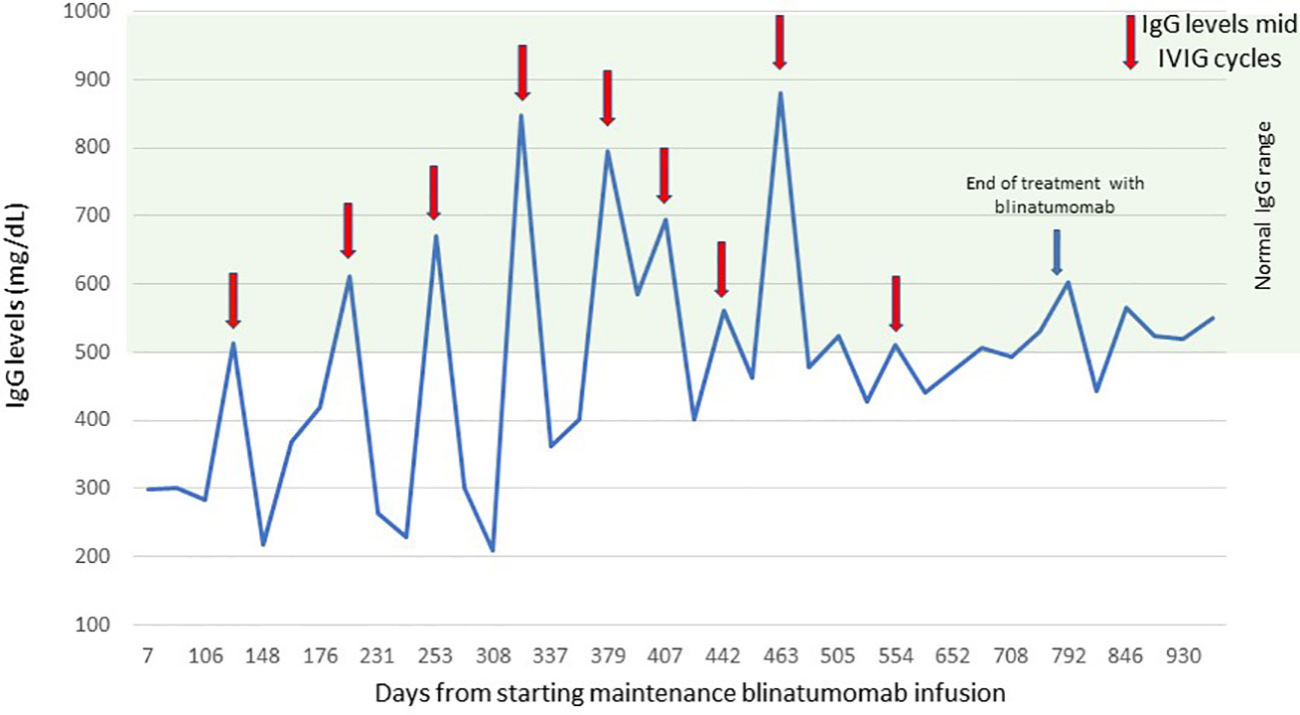
Serum IgG levels. The figure shows the level of serum IgG during blinatumomab infusions and 6 months after treatment. IgG, immunoglobulin G. Red arrows indicate IgG levels mid-IVIG cycles, the blue arrow indicates the date of end of treatment with blinatumomab.

The patient and the family were compliant with the blinatumomab infusions and agreed to receive blinatumomab instead of continuing conventional chemotherapy. It resulted in infrequent hospitalizations or ER visits. The patient tolerated the blinatumomab infusions well. There was one episode of pneumonia during cycle 1 of consolidation and *Staphylococcus epidermidis* sepsis with bilateral pneumonia during cycle 1 of maintenance with blinatumomab, which could also be attributed to an underlying incompletely treated bacterial or fungal infections and prolonged intubation. Blinatumomab can also cause severe neutropenia and hypogammaglobulinemia during the treatment (21). Our patient had grade 4 neutropenia and leucopenia during the first cycle of blinatumomab consolidation, which recovered in 2 weeks. Wo et al. conducted a small sample size study indicating no difference in secondary hypogammaglobinemia and associated infection risk when two cycles of blinatumomab were used (22, 23). However, since we planned blinatumomab for long-term treatment and the paucity of data in children, we administered monthly IVIG. The immunoglobulin G (IgG) troughs during blinatumomab infusions always remained below the normal level; however, mid-IVIG cycle levels were within the normal range (**Figure 2**). Zugmaier et al. showed that the IgG recovery after blinatumomab exceeded 50% of the baseline in three out of five responders in adults (23). In our patient, we were able to wean the IVIG infusions to every 6 weeks 1 year post-blinatumomab therapy to maintain troughs around normal levels.

During cycle 11 of consolidation, she had an episode of grade I seizure lasting 5 seconds. This could be due to neurotoxicity from intrathecal methotrexate that she received 11 days prior or due to blinatumomab. Pediatric patients with methotrexate neurotoxicity when rechallenged with intrathecal methotrexate tolerated it well, and blinatumomab neurotoxicity was reversible in pediatric clinical trials (11, 24). The patient was started on Keppra and tolerated future intrathecal methotrexate and blinatumomab without any other episodes of neurotoxicity.

In summary, we present the first case to our knowledge for demonstrating that the use of blinatumomab therapy upfront for two cycles of consolidation and 2 years of maintenance therapy in a high-risk B-ALL pediatric patient is feasible and well tolerated, leading to sustained remission. Our patient remains in remission at 25 months off treatment at the time of publication without any further bridging transplant or chemotherapy. Blinatumomab can be used as a salvage option for pediatric patients with severe toxicities due to conventional chemotherapies causing delay and early termination of treatment with significant morbidity and mortality leading to high chances of relapse in high-risk B-ALL patients. However, this is a case report and further studies are required to determine the appropriate length of blinatumomab therapy along with long-term remission rates and adverse effects.

In conclusion, blinatumomab given upfront as consolidation and maintenance therapy for 2 years in a pediatric high-risk B-ALL patient with significant toxicities to conventional chemotherapy was feasible and well tolerated without any irreversible toxicity and led to sustained remission without any bridging transplant or further chemotherapy.

## Data availability statement

The original contributions presented in the study are included in the article/supplementary material. Further inquiries can be directed to the corresponding author.

## Ethics statement

Written informed consent was obtained from the patient/family for publication of data included in this article.

## Author contributions

SG, JC, and JL were involved in the writing and preparation of this manuscript. All authors read and approved this manuscript.

## References

[1] Salzer WL Burke MJ Devidas M Chen S Gore L Larsen EC. Toxicity associated with intensive postinduction therapy incorporating clofarabine in the very high-risk stratum of patients with newly diagnosed high-risk B-lymphoblastic leukemia: A report from the Children's Oncology Group study AALL1131. Cancer (2018) 124(6):1150–9. doi: 10.1002/cncr.31099 PMC5839964 29266189

[2] Pui CH Yang JJ Hunger SP Pieters R Schrappe M Biondi A. Childhood acute lymphoblastic leukemia: progress through collaboration. J Clin Oncol (2015) 33(27):2938–48. doi: 10.1200/JCO.2014.59.1636 PMC4567699 26304874

[3] Moricke A Zimmermann M Reiter A Henze G Schrauder A Gadner H. Long-term results of five consecutive trials in childhood acute lymphoblastic leukemia performed by the ALL-BFM study group from 1981 to 2000. Leukemia (2010) 24(2):265–84. doi: 10.1038/leu.2009.257 20010625

[4] Mulrooney DA Hyun G Ness KK Bhakta N Pui CH Ehrhardt MJ. The changing burden of long-term health outcomes in survivors of childhood acute lymphoblastic leukaemia: a retrospective analysis of the St Jude Lifetime Cohort Study. Lancet Haematol (2019) 6(6):e306–e16. doi: 10.1016/S2352-3026(19)30050-X PMC6756152 31078468

[5] Larsen EC Devidas M Chen S Salzer WL Raetz EA Loh ML. Dexamethasone and high-dose methotrexate improve outcome for children and young adults with high-risk B-acute lymphoblastic leukemia: A report from children's oncology group study AALL0232. J Clin Oncol (2016) 34(20):2380–8. doi: 10.1200/JCO.2015.62.4544 PMC4981974 27114587

[6] Oh SM Byun JM Chang E Kang CK Shin DY Koh Y. Incidence of invasive fungal infection in acute lymphoblastic and acute myelogenous leukemia in the era of antimold prophylaxis. Sci Rep (2021) 11(1):22160. doi: 10.1038/s41598-021-01716-2 34773060 PMC8590008

[7] Fulbright JM Raman S McClellan WS August KJ. Late effects of childhood leukemia therapy. Curr Hematol Malig Rep (2011) 6(3):195–205. doi: 10.1007/s11899-011-0094-x 21695425

[8] van der Does-van den Berg A de Vaan GA van Weerden JF Hahlen K van Weel-Sipman M Veerman AJ. Late effects among long-term survivors of childhood acute leukemia in The Netherlands: a Dutch Childhood Leukemia Study Group Report. Pediatr Res (1995) 38(5):802–7. doi: 10.1203/00006450-199511000-00027 8552452

[9] Kantarjian H Stein A Gokbuget N Fielding AK Schuh AC Ribera JM. Blinatumomab versus chemotherapy for advanced acute lymphoblastic leukemia. N Engl J Med (2017) 376(9):836–47. doi: 10.1056/NEJMoa1609783 PMC5881572 28249141

[10] von Stackelberg A Locatelli F Zugmaier G Handgretinger R Trippett TM Rizzari C. Phase I/phase II study of blinatumomab in pediatric patients with relapsed/refractory acute lymphoblastic leukemia. J Clin Oncol (2016) 34(36):4381–9. doi: 10.1200/JCO.2016.67.3301 27998223

[11] Brown PA Ji L Xu X Devidas M Hogan LE Borowitz MJ. Effect of postreinduction therapy consolidation with blinatumomab vs chemotherapy on disease-free survival in children, adolescents, and young adults with first relapse of B-cell acute lymphoblastic leukemia: A randomized clinical trial. JAMA (2021) 325(9):833–42. doi: 10.1001/jama.2021.0669 PMC7926290 33651090

[12] Locatelli F Zugmaier G Rizzari C Morris JD Gruhn B Klingebiel T. Effect of blinatumomab vs chemotherapy on event-free survival among children with high-risk first-relapse B-cell acute lymphoblastic leukemia: A randomized clinical trial. JAMA (2021) 325(9):843–54. doi: 10.1001/jama.2021.0987 PMC7926287 33651091

[13] Hunger SP Lu X Devidas M Camitta BM Gaynon PS Winick NJ. Improved survival for children and adolescents with acute lymphoblastic leukemia between 1990 and 2005: a report from the children's oncology group. J Clin Oncol (2012) 30(14):1663–9. doi: 10.1200/JCO.2011.37.8018 PMC3383113 22412151

[14] Fisher BT Robinson PD Lehrnbecher T Steinbach WJ Zaoutis TE Phillips B. Risk factors for invasive fungal disease in pediatric cancer and hematopoietic stem cell transplantation: A systematic review. J Pediatr Infect Dis Soc (2018) 7(3):191–8. doi: 10.1093/jpids/pix030 PMC12427443 28549148

[15] Mody R Li S Dover DC Sallan S Leisenring W Oeffinger KC. Twenty-five-year follow-up among survivors of childhood acute lymphoblastic leukemia: a report from the Childhood Cancer Survivor Study. Blood (2008) 111(12):5515–23. doi: 10.1182/blood-2007-10-117150 PMC2424150 18334672

[16] Angiolillo AL Schore RJ Kairalla JA Devidas M Rabin KR Zweidler-McKay P. Excellent outcomes with reduced frequency of vincristine and dexamethasone pulses in standard-risk B-lymphoblastic leukemia: results from children's oncology group AALL0932. J Clin Oncol (2021) 39(13):1437–47. doi: 10.1200/JCO.20.00494 PMC8274746 33411585

[17] Litzow MR Sun Z Paietta E Mattison RJ Lazarus HM Rowe JM. Consolidation therapy with blinatumomab improves overall survival in newly diagnosed adult patients with B-lineage acute lymphoblastic leukemia in measurable residual disease negative remission: results from the ECOG-ACRIN E1910 randomized phase III national cooperative clinical trials network trial. Blood (2022) 140(Supplement 2). LBA-1-LBA. doi: 10.1182/blood-2022-171751

[18] Gokbuget N Dombret H Bonifacio M Reichle A Graux C Faul C. Blinatumomab for minimal residual disease in adults with B-cell precursor acute lymphoblastic leukemia. Blood (2018) 131(14):1522–31. doi: 10.1182/blood-2017-08-798322 PMC6027091 29358182

[19] Topp MS Gokbuget N Zugmaier G Degenhard E Goebeler ME Klinger M. Long-term follow-up of hematologic relapse-free survival in a phase 2 study of blinatumomab in patients with MRD in B-lineage ALL. Blood (2012) 120(26):5185–7. doi: 10.1182/blood-2012-07-441030 23024237

[20] Topp MS Kufer P Gokbuget N Goebeler M Klinger M Neumann S. Targeted therapy with the T-cell-engaging antibody blinatumomab of chemotherapy-refractory minimal residual disease in B-lineage acute lymphoblastic leukemia patients results in high response rate and prolonged leukemia-free survival. J Clin Oncol (2011) 29(18):2493–8. doi: 10.1200/JCO.2010.32.7270 21576633

[21] Maschmeyer G De Greef J Mellinghoff SC Nosari A Thiebaut-Bertrand A Bergeron A. Infections associated with immunotherapeutic and molecular targeted agents in hematology and oncology. A position paper by the European Conference on Infections in Leukemia (ECIL). Leukemia (2019) 33(4):844–62. doi: 10.1038/s41375-019-0388-x PMC6484704 30700842

[22] Wo S Levavi H Mascarenhas J Kremyanskaya M Navada S Bar-Natan M. Immunoglobulin repletion during blinatumomab therapy does not reduce the rate of secondary hypogammaglobulinemia and associated infectious risk. Blood Res (2022) 57(2):135–43. doi: 10.5045/br.2022.2021163 PMC9242831 35551109

[23] Zugmaier G Topp MS Alekar S Viardot A Horst HA Neumann S. Long-term follow-up of serum immunoglobulin levels in blinatumomab-treated patients with minimal residual disease-positive B-precursor acute lymphoblastic leukemia. Blood Cancer J (2014) 4(9):244. doi: 10.1038/bcj.2014.64 25192414 PMC4183773

[24] Bhojwani D Sabin ND Pei D Yang JJ Khan RB Panetta JC. Methotrexate-induced neurotoxicity and leukoencephalopathy in childhood acute lymphoblastic leukemia. J Clin Oncol (2014) 32(9):949–59. doi: 10.1200/JCO.2013.53.0808 PMC3948096 24550419

